# Supplementary material for: Resolving the full spectrum of human genome variation using Linked-Reads
Source: Genome Res. 2019 Apr;29(4):635–45. doi: 10.1101/gr.234443.118 (PMC6442396; doi:10.1101/gr.234443.118)
Supplement: Supplemental Material [file supp_gr.234443.118_Supplemental_longranger-2.2.zip › longranger-2.2.tar.bz2!/lr2.2-public/lib/stan/cmdstan/stan/doc/api/html/math_2matrix_2softmax_8hpp_source.html]

Stan: src/stan/math/matrix/softmax.hpp Source File


|  |  |
| --- | --- |
|  | Stan  2.5.0  probability, sampling & optimization |


- Main Page
- Related Pages
- Namespaces
- Classes
- Files

- File List
- File Members

All Classes Namespaces Files Functions Variables Typedefs Enumerations Enumerator Friends Macros Pages

- src
- stan
- math
- matrix

softmax.hpp

Go to the documentation of this file.

1 #ifndef STAN\_\_MATH\_\_MATRIX\_\_SOFTMAX\_HPP

2 #define STAN\_\_MATH\_\_MATRIX\_\_SOFTMAX\_HPP

3

4 #include <stan/math/error\_handling/matrix/check\_nonzero\_size.hpp>

5 #include <cmath>

6 #include <stan/math/matrix/Eigen.hpp>

7

8 namespace stan {

9  namespace math {

10

44  template <typename T>

45  inline Eigen::Matrix<T,Eigen::Dynamic,1>

46  softmax(const Eigen::Matrix<T,Eigen::Dynamic,1>& v) {

47  using std::exp;

48  stan::math::check\_nonzero\_size("softmax(%1%)",v,"v",(double\*)0);

49  Eigen::Matrix<T,Eigen::Dynamic,1> theta(v.size());

50  T sum(0.0);

51  T max\_v = v.maxCoeff();

52  for (int i = 0; i < v.size(); ++i) {

53  theta(i) = exp(v(i) - max\_v); // extra work for (v[i] == max\_v)

54  sum += theta(i); // extra work vs. sum() w. auto-diff

55  }

56  for (int i = 0; i < v.size(); ++i)

57  theta(i) /= sum;

58  return theta;

59  }

60

61  }

62 }

63 #endif

stan::math::softmax

Eigen::Matrix< T, Eigen::Dynamic, 1 > softmax(const Eigen::Matrix< T, Eigen::Dynamic, 1 > &v)

Return the softmax of the specified vector.

**Definition:** softmax.hpp:46

stan::math::exp

Eigen::Matrix< T, Rows, Cols > exp(const Eigen::Matrix< T, Rows, Cols > &m)

Return the element-wise exponentiation of the matrix or vector.

**Definition:** exp.hpp:18

Eigen.hpp

stan::math::sum

double sum(std::vector< double > &x)

**Definition:** sum.hpp:10

check\_nonzero\_size.hpp

stan::agrad::exp

fvar< T > exp(const fvar< T > &x)

**Definition:** exp.hpp:16

stan::math::check\_nonzero\_size

bool check\_nonzero\_size(const char \*function, const T\_y &y, const char \*name, T\_result \*result)

Return true if the specified matrix/vector is of non-zero size.

**Definition:** check\_nonzero\_size.hpp:33


---

[ Stan Home Page ]

*© 2011–2014,
Stan Development Team.*
